# A study on cadmium immobilization by bacterial consortia: *Achromobacter insuavis* SL8 and *Enterobacter cancerogenus* SL12

**DOI:** 10.1128/spectrum.01081-26

**Published:** 2026-06-17

**Authors:** Yu Tao, Shengying Ji, Chi Zhou, Di Peng, Xin Li

**Affiliations:** 1 Hunan Academy of Agricultural Sciences 441102, Changsha, Hunan, China; 2 Hunan Institute of Microbiology 639864, Changsha, Hunan, China; 3 Hunan Engineering Research Center of Endophytic Microbial Resources Exploration and utilization in Plants 58287 , Changsha, Hunan, China; 4 Hunan Institute of Vegetable Research, Changsha, Hunan, China; University of Minnesota Twin Cities, St. Paul, Minnesota, USA

**Keywords:** microbial cadmium immobilization, Cd-resistant bacteria, integrated multi-technique analysis, mechanisms of Cd resistance

## Abstract

Microbial bioremediation represents one of the most environmentally sustainable strategies for mitigating cadmium (Cd) pollution. This study employed an integrated approach combining spectroscopy, metabolomics, and proteomics to elucidate the molecular mechanisms underlying Cd detoxification in *Achromobacter insuavis* SL8 and *Enterobacter cancerogenus* SL12. The results demonstrated that both strains exhibit high Cd tolerance, with minimum inhibitory concentrations (MICs) of 600 mg/L and 400 mg/L for SL8 and SL12, respectively. In a 100 mg/L Cd solution, the strains achieved removal rates of 69.5% (SL8) and 70.8% (SL12), which increased to 80.9% under co-culture conditions. Spectroscopic analyses revealed that the co-culture facilitated Cd detoxification through the formation of CdS precipitates and modifications of key surface functional groups (phosphate, N-H, and C=O groups). Metabolomic and proteomic profiling indicated significant up-regulation of metabolites and proteins involved in metal transport, biosorption, chelation, energy metabolism, and antioxidant defense, collectively maintaining cellular Cd homeostasis. To validate field applicability, pot experiments showed Cd removal rates of 13% in soil and 20% in Chinese cabbage. This multi-omics investigation not only deepens the understanding of microbial adaptive responses to Cd stress, but also provides insights for developing novel microbial consortia for efficient and eco-friendly bioremediation of Cd-contaminated environments.

**IMPORTANCE:**

Cadmium contamination in agricultural soils poses serious risks to food safety and human health. Despite being a highly sustainable approach for mitigating cadmium pollution, microbial bioremediation still lacks practical and effective solutions. In this study, we reveal how a two-member bacterial consortium (*Achromobacter insuavis* SL8 and *Enterobacter cancerogenus* SL12) works together to immobilize Cd through complementary mechanisms. Through a multi-omics and spectroscopic approach, we discovered that the consortium not only outperforms individual strains in cadmium removal but also enhances plant growth while decreasing cadmium uptake in crops. These findings provide a scientific basis for developing effective bioremediation strategies using microbial partnerships. Our work advances the understanding of how bacteria cooperatively respond to heavy metal stress and offers a promising, eco-friendly approach to remediate cadmium-polluted soils, ultimately contributing to safer food production and improved environmental health.

## INTRODUCTION

Cadmium (Cd) pollution poses escalating environmental and public health threats, mobilizing urgent global scientific attention (1). Owing to its notable mobility and toxicity, Cd remains a pervasive environmental contaminant even at trace concentrations (2). Originating from agricultural runoff, industrial discharges, and mining operations, Cd accumulation in water and soil systems jeopardizes human health, ecological stability, and agricultural sustainability (3). Particularly concerning is Cd’s impact on agricultural systems, where it impairs essential plant physiological processes (e.g., seed germination and root elongation), directly compromising food safety and security (4). Addressing the environmental footprint of Cd requires the development of innovative, efficient, and sustainable remediation technologies.

Among various remediation strategies, microbial bioremediation has emerged as a promising approach, presenting a sustainable and ecologically sound alternative to conventional methods. These methods often rely on invasive physical excavation or chemical treatments—practices that are cost-prohibitive and risk generating secondary pollutants (5, 6). Microorganisms leverage their innate capabilities to adsorb, metabolize, and sequester heavy metals, thereby diminishing the bioavailability and toxicity of Cd in contaminated environments (7).

Despite the potential of microbial bioremediation, the deployment of mixed microbial consortia as a deliberate strategy for Cd pollution remediation remains underexplored. Preliminary studies suggest that microbial consortia may exhibit synergistic effects, surpassing single-strain approaches in remediation capabilities (8). For example, consortia have demonstrated promising potential in reducing extractable Cd at contaminated sites, suggesting enhanced bioremediation outcomes (9). However, the mechanistic basis for these synergistic interactions, particularly those enhancing Cd immobilization, remains unclear. This knowledge gap underscores the need for deeper investigation into the interactive dynamics and mechanistic underpinnings of microbial consortia in Cd bioremediation.

Building on this premise, our study investigates the combined effects of *Achromobacter insuavis* SL8 and *Enterobacter cancerogenus* SL12, two strains with documented Cd resistance and bioremediation potential (10, 11). While their individual bioremediation capabilities have been characterized, the interactive dynamics within a co-culture system remain unexplored, particularly their collective impact on Cd immobilization. This study aims to elucidate how the synergistic interaction between *A. insuavi*s SL8 and *E. cancerogenus* SL12 enhances Cd immobilization through physicochemical, metabolic, and multi-omics analyses. By delving into these aspects, we seek to advance the understanding of microbial consortia in heavy metal bioremediation and facilitate the development of more effective and sustainable remediation strategies.

## MATERIALS AND METHODS

### Collection, isolation, and identification of strain

Soil samples were collected from Cd-contaminated areas around a mine in Liuyang, Hunan Province, China (28°2′N, 113°64′E). The comprehensive procedure for bacterial isolation and identification is provided in the Supplementary Information (SI available at https://zenodo.org/records/20263325). Samples were collected from the top 20 cm of the soil surface in five randomized replicates, each weighing approximately 250 g. Part of each sample was refrigerated at 4℃ for subsequent microbial isolation, while the remainder was air-dried and sieved for physicochemical characterization. Microbial enrichment was carried out in LB liquid medium consisting of 10 g tryptone, 10 g NaCl, and 5 g yeast extract per liter of water, adjusted to pH of 7.0. Following enrichment, the medium was serially diluted with sterile water and plated on LB agar plates supplemented with 100 mg/L Cd^2+^ (CdCl_2_⋅2.5H_2_O) to facilitate strain isolation.

### Tolerance and removal performance of the strains to Cd

The determination of minimum inhibitory concentration (MIC) for previously identified strains was performed (12). Firstly, a predetermined volume of strain culture was inoculated into 100 mL Erlenmeyer flasks containing 50 mL of LB culture medium (pH 7.0) to attain an initial optical density (OD600) of approximately 0.2. Then, a filter-sterilized 50 mM solution of Cd was added to these flasks, resulting in initial Cd concentrations ranging from 0 to 1 g/L. Finally, the cultures were incubated at 37℃ with shaking at 180 rpm in a horizontal shaker. Culture samples (1 mL) were collected every 2 h to measure OD600, monitoring microbial growth dynamics.

Growth curves of *A. insuavis* SL8 and *E. cancerogenu*s SL12 under Cd exposure were subsequently plotted. Purified strains of *A. insuavis* SL8 and *E. cancerogenus* SL12 were cultured in 100 mL liquid LB medium at 30℃ with shaking at 200 rpm. The experiment featured three treatments, namely “SL8,” “SL12,” and “SL8 + SL12.” When the bacterial suspension reached an OD600 of 0.8, the bacterial culture was in the late exponential growth phase. 1% (vol/vol) of the culture was inoculated into 30 mL of LB medium supplemented with 0, 50, and 100 mg/L Cd^2+^, respectively. The inoculated cultures were then incubated under identical conditions (30℃, 200 rpm). Culture samples (0.5 mL) were collected at predetermined intervals (0, 6, 24, 30, 54, and 70 h post-inoculation), and the bacterial concentrations were determined using a light microscope and counted with a hemocytometer.

To evaluate the Cd removal performance of the strain, "SL8," "SL12," and "SL8 + SL12" strains were inoculated into 100 mL of LB liquid medium and incubated at 30℃ with shaking at 200 rpm. When the bacterial suspension reached an OD600 of 0.8, 1% (vol/vol) of the culture was transferred into liquid LB medium supplemented with 50 mg/L or 100 mg/L Cd^2+^, followed by incubation under identical conditions (30℃, 200 rpm). Culture samples (1.5 mL) were collected on days 0, 1, 3, and 7, centrifuged at 12,000 rpm for 10 min, and 1 mL of supernatant was filtered through a 0.22 μm membrane filter. The concentration of Cd^2+^ in the filtrate was determined using atomic absorption spectroscopy. The Cd removal efficiency (RE-Cd) was calculated using the formula RE-Cd (%) = (C₀ – Cₜ)/C₀ × 100 from Fathollahi and Coupe (13), based on the Cd²^+^ concentration in the supernatant on day 7, with an initial Cd concentration of 100 mg/L in LB medium. The other tested strains were all evaluated for RE-Cd under the same conditions. The contributions of Cd immobilization by the strains through extracellular adsorption, intracellular accumulation, and bioprecipitation were quantified according to the method described by Han et al. (14), using an initial Cd concentration of 100 mg/L in LB medium and a 7-day incubation period (14).

### Characterization of precipitates

After a 7-day incubation for Cd removal, cultures were centrifuged to collect bacterial precipitates for characterization. Analytical techniques included scanning electron microscopy with energy-dispersive X-ray spectroscopy (SEM-EDX), transmission electron microscopy (TEM), X-ray photoelectron spectroscopy (XPS), and Fourier transform infrared (FTIR) spectroscopy to analyze Cd immobilization sites and mechanisms (15, 16).

### Combined metabolomics and proteomics analysis

In this study, bacterial strains “SL8,” “SL12,” and the co-cultured “SL8 + SL12” were cultivated in LB medium supplemented with or without 100 mg/L of Cd^2+^ for 48 h. Six replicates per treatment were prepared to ensure statistical robustness. Subsequently, the cultures were centrifuged at 10,000 rpm to separate supernatant from the cell pellet. The extracellular metabolites in the supernatant were extracted following the metabolomic analysis provided by Biomec’s protocols. Briefly, 100 μL of supernatant was mixed with 500 μL of a 1:1 methanol-acetonitrile solution, vortexed for 30 s, and ultrasonic treatment for 10 min in an ice water bath. Samples were incubated at −20℃ for one h before centrifugation at 4℃. 500 μL of the supernatant was then removed and dried using a vacuum concentrator, and the dry metabolite extract was redissolved. This reconstituted solution was vortexed for 30 s, subjected to a second round of ultrasonic treatment for 10 min, and centrifuged at 12,000 rpm for 15 min at 4℃. A precise 120 μL aliquot of this supernatant was transferred into a 2 mL injection bottle, with 10 μL of each sample pooled into quality control samples for subsequent online detection.

For proteomic analysis (17), extracellular proteins were quantified using a label-free assay. Cells were lysed with a buffer containing 7 M urea, 2 M thiourea, and 0.1% CHAPS, then homogenized with a tissue grinder (lysate to protease inhibitor ratio 50:1) and subjected to ultrasonic disruption. After centrifugation at 14,000 × g for 30 min, the supernatant was discarded, and the protein-rich precipitate was retained for analysis.

Protein and metabolite samples were analyzed using an Agilent 1290 Infinity LC system for ultra-high-performance liquid chromatography, coupled with a TripleTOF 5600 mass spectrometer for precise measurement. Electrospray ionization (ESI) facilitated the detection of both positively and negatively charged ions. Original data were converted into MzXML format via ProteoWizard and further processed with the XCMS program for peak alignment, retention time correction, and peak area quantification. Metabolite identification was confirmed by accurate mass matching (<25 ppm) and secondary spectra comparison.

In order to illuminate the cooperative Cd detoxification capabilities of SL8 and SL12 strains through an integrated metabonomic and proteomic approach, the experiment was structured to assess the impact of Cd exposure on these consortia by comparing groups subjected to Cd^2+^ (100 mg/L) against controls without such exposure. The groups were delineated as follows: SL8 strains under Cd^2+^ stress (group A), SL12 strains under Cd^2+^ stress (group B), co-cultured SL8 and SL12 without Cd^2+^ (group C), and co-cultured SL8 and SL12 under Cd^2+^ stress (group D). This design provided a comprehensive view of how co-cultivation and metal stress affected microbial protein expression and metabolic pathways.

### Pot experiments

The influence of co-cultured strains, *A. insuavis* SL8 and *E. cancerogenus* SL12, on the growth of Chinese cabbage (*Brassica campestris L*.) was meticulously assessed through a series of pot experiments. The soil used was collected from an agricultural field in Changsha City, Hunan Province, China (113°58.5′E, 28°30.473′N). Basic physicochemical characteristics of soil used for pot experiments are shown in Table 1. The initial Cd concentration in the soil was determined to be 0.708 mg/kg. To establish a range of Cd contamination levels, CdCl_2_ was methodically added to the soil at concentrations of 0, 5, and 10 mg/kg, corresponding to control, moderate, and highly contaminated levels, respectively. After the addition of CdCl_2_, the soil samples were thoroughly homogenized and allowed to equilibrate for 14 days. Each treatment was conducted in quintuplicate.

**TABLE 1:** Basic physicochemical characteristics of soil used for greenhouse pot experiments with Chinese cabbage (*Brassica campestris L*.)^*a*^^,^^*b*^

Physicochemical characteristics	Content
pH (soil: water = 1:2)	5.80
Soil organic matter (g·kg^−1^)	21.85
Total N (g·kg^−1^)	1.39
Total *P* (g·kg^−1^)	2.08
Total K (g·kg^−1^)	11.74
Available *P* (mg·kg^−1^)	385.40
Available K (mg·kg^−1^)	424.00
Total Cd (mg·kg^−1^)	0.71
Total Cr (mg·kg^−1^)	64.40
Total Pd (mg·kg^−1^)	38.40
Total As (mg·kg^−1^)	11.70

^*a*^ The latitude and longitude of the soil collection site are 113°58.5′E, 28°30.473′N.

^*b*^ Total N, total nitrogen; Total P, total phosphorus; Total K, total potassium; Available P, available phosphorus; Available K, available potassium; Total Cd, total cadmium; Total Cr, total chromium; Total Pb, total lead; Total As, total arsenic.

The duration of the pot experiment was 30 days, and each pot (23 cm diameter, 16 cm height) was filled with 5.0 kg of the prepared soil. Bacterial inoculation was performed by introducing the bacterial strains at a concentration of 2 × 10^7^ CFU/g soil to each pot. After inoculation, the soil was allowed to stabilize for 4 days before planting. Subsequently, the plants were cultivated in a greenhouse with controlled conditions of 30.0 ± 2°C during the day and 20.0 ± 2°C during the night with a 12-h photoperiod. Throughout the cultivation period, soil moisture was maintained at optimal levels.

Upon harvesting, the wet weight and Cd content of both the root and aerial parts of the plants were measured. Additionally, rhizosphere soil samples were collected for Cd speciation analysis and 16S rRNA metagenomic sequencing. Cd speciation analysis was performed using a sequential extraction method (18). Metagenomic analysis was conducted on rhizosphere soils from the pots with moderate Cd contamination (5 mg/kg) on days 0, 14, and 30. DNA extraction and subsequent analysis were performed according to established protocols (19). Principal Coordinate Analysis (PCoA) was performed using R packages (package ade4, version 3.1.1; http://www.r-project.org).

### Statistical analysis

The data regarding the soil physicochemical properties were expressed as mean ± standard deviation. All figures were generated using Origin Pro 8.0, ensuring clear and accurate presentation of the experimental results.

## RESULTS AND DISCUSSION

### Identification and growth kinetics of Cd-resistant strains SL8 and SL12

**Fig 1:**
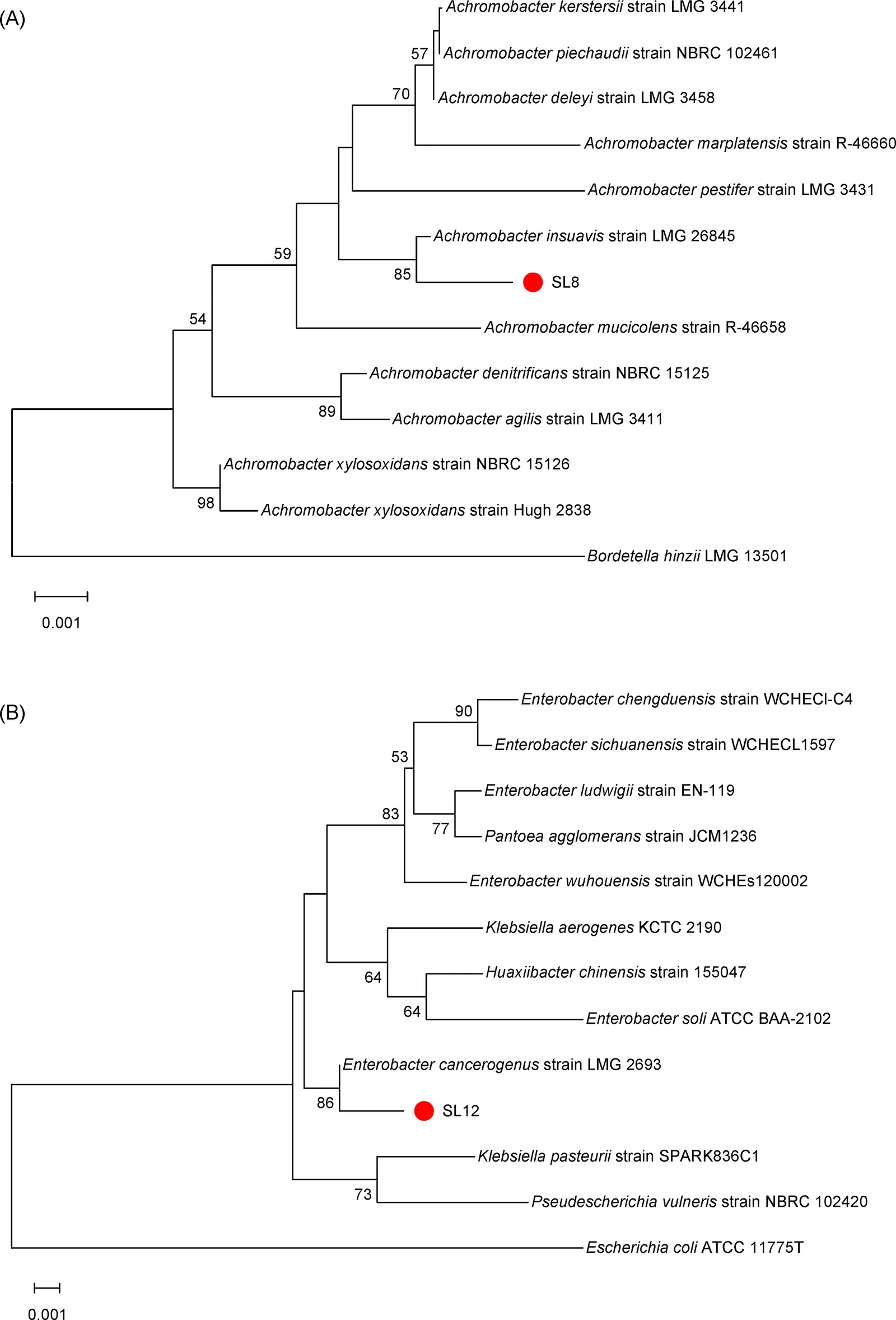
Based on 16S rRNA gene sequences, a neighbor-joining tree showed the phylogenetic positions of isolated Cd-resistant strains, SL8 and SL12. Neighbor-joining trees based on 16S rRNA gene sequences showing the phylogenetic positions of isolated Cd-resistant strains. (A) Phylogenetic tree of strain SL8; (B) Phylogenetic tree of strain SL12.

Based on 16S rRNA gene sequence analysis, SL8 was identified as *Achromobacter* sp., and SL12 as *Enterobacter* sp., with sequence homologies of 99.79% and 99.31% (Fig. 1), respectively. Notably, the strains' MICs, determined at 600 mg/L for SL8 and 400 mg/L for SL12 (Table 2), significantly surpass those of other Cd-resistant bacteria, indicative of their innate capabilities for Cd resistance and removal.

**TABLE 2:** Cd(II) resistance levels and immobilization abilities of bacterial strains isolated from Cd-contaminated soil

Bacterial strains	MIC^*a*^ of Cd(II) (mg/L)	Cd removal efficiency (%)
*Pseudomonas* sp.SL2	200	15.37 ± 2.02
*Enterobacter* sp. SL3	100	18.56 ± 0.04
*Enterobacter* sp. SL4	200	38.19 ± 1.33
*Cupriavidus* sp. SL5	200	26.38 ± 2.46
*Bacillus* sp. SL6	600	30.88 ± 0.89
*Alcaligenes* sp. SL7	400	67.77 ± 0.06
*Achromobacter* sp. SL8	600	69.55 ± 0.21
*Pseudarthrobacter* sp. SL9	200	12.94 ± 1.90
*Paenibacillus* sp. SL10	200	23.43 ± 0.15
*Serratia* sp. SL11	600	62.02 ± 0.10
*Enterobacter* sp. SL12	400	70.81 ± 0.10
*Enterobacter* sp. SL14	200	55.77 ± 0.30
*Enterobacter* sp. SL15	200	40.04 ± 1.21
*Paenibacillus* sp. gs1	200	20.56 ± 1.35
*Achromobacter* sp.SL8 + *Enterobacter* sp. SL12	600	80.90 ± 0.23
Other strains combinations	200–600	10–70

^*a*^ To establish bacterial resistance level of Cd(II), minimum inhibitory concentration (MIC) tests were performed in LB liquid medium at 30°C for 48 h. Results are in the form mean ± standard deviation, *n* = 3.

**Fig 2:**
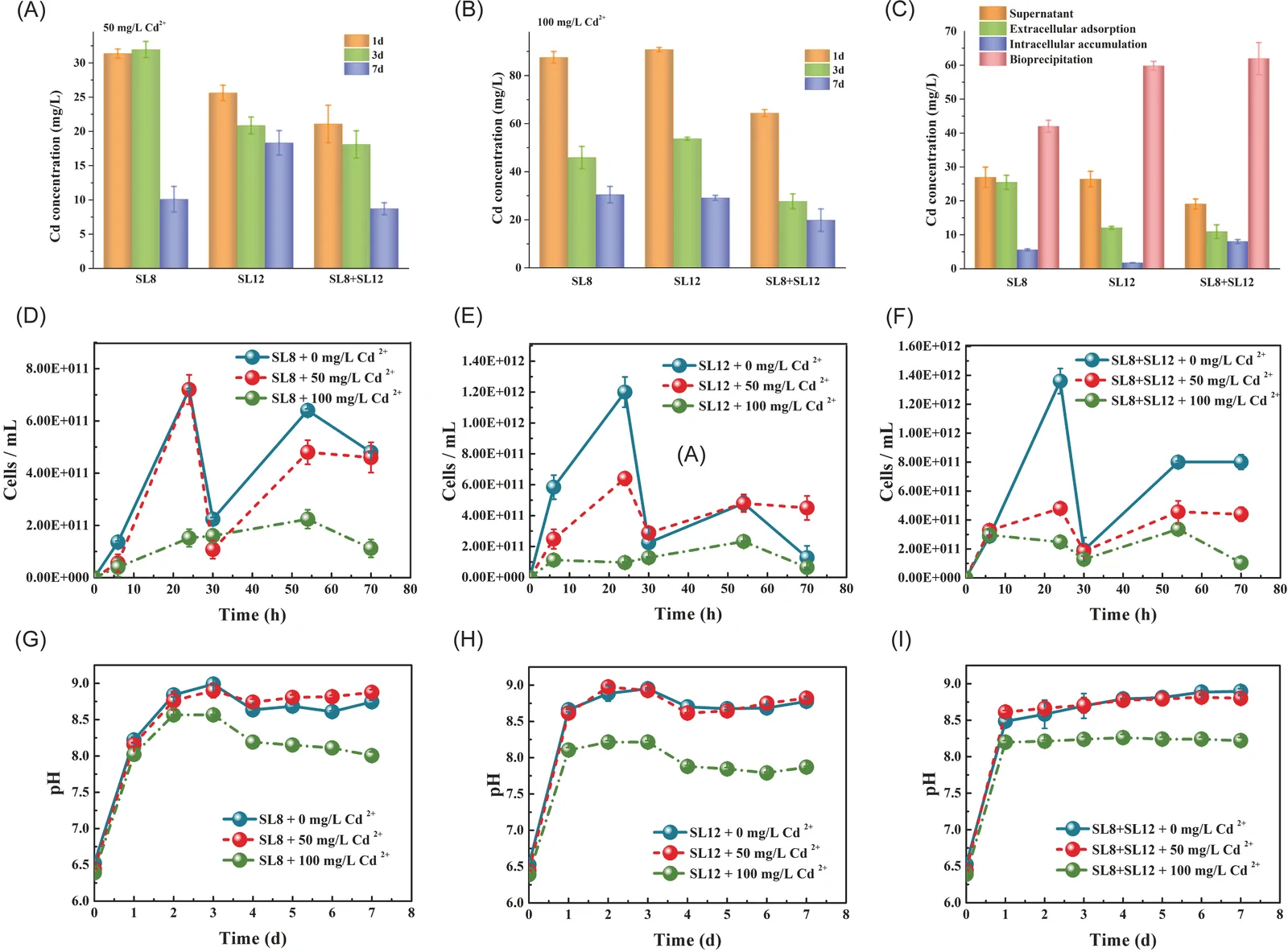
The adsorption capacity of SL8, SL12, and cocultured strains grown in culture media supplemented with increasing concentrations of Cd ions, including (**A**) 50 mg/L Cd^2+^, (**B**) 100 mg/L Cd^2+^, and (**C**) 100 mg/L Cd^2+^. Growth curves (**D–F**) and pH (**G–I**) changes of SL8, SL12, and cocultured strains of SL8 and SL12 at three concentrations. Error bars indicate standard deviations of the means (*n* = 3).

These findings underscore the strains' strong resistance profiles and their effectiveness in Cd removal, as demonstrated by high RE-Cd and growth kinetics under Cd stress (Fig. 2A, B, D, E, and F), confirming their potential as promising candidates for environmental Cd remediation efforts. Despite their overall resilience to Cd exposure, a concentration-dependent suppression of growth efficiency was observed.

### Growth inhibition and cadmium removal efficiency

This study examined RE-Cd of both individual and co-cultured strains SL8 and SL12 across varying Cd concentrations. The results revealed a complex interplay of biological and chemical factors enhancing Cd detoxification. Notably, the RE-Cd for all tested configurations increased gradually over time (Fig. 2A and B), a trend strongly associated with changes in environmental pH. The pH increased sharply from approximately 6.5 to 8.0–8.5 within the first 2 days of incubation and then stabilized (Fig. 2G through I). This pH elevation is instrumental in enhancing the competitiveness of metal ions for bacterial binding sites and promoting hydroxide precipitation, thereby facilitating Cd^2+^ removal (20). After a 7-day incubation period, RE-Cd values reached 82.6% and 69.55% for SL8, 63.4% and 70.8% for SL12, and 82.6% and 80.9% for the co-cultured strains at initial Cd concentrations of 50 mg/L and 100 mg/L, respectively. These findings underscore the distinctive responses of SL8 and SL12 to Cd stress. Specifically, SL8 exhibited a decrease in RE-Cd under higher Cd stress, consistent with previous reports that elevated Cd levels enhance cellular toxicity and reduce biosorption capacity (21, 22). Conversely, SL12 demonstrated an 11.6% improvement in RE-Cd with increasing Cd concentration, suggesting an adaptive energy conservation strategy that enhances activity against metal toxicity. This resilience is echoed in prior studies in which metal-resistant microbes under stress conditions down-regulate tricarboxylic acid (TCA) cycle and nitrogen metabolism pathways to reinforce detoxification and resistance mechanisms (9, 22). Remarkably, the co-cultured "SL8 + SL12" strains exhibited a synergistic effect, enhancing RE-Cd across all tested Cd concentrations. This synergy suggests a more stable equilibrium in the incubation solution, likely due to optimized pH conditions and altered Cd removal pathways, thus presenting a promising approach for Cd bioremediation (Fig. 2A, B, C, G through I).

Analysis of Cd removal mechanisms revealed a predominance of bioprecipitation in Cd detoxification, consistent under both individual and co-cultured conditions (Fig. 2C). XPS analysis further confirmed the formation of CdS precipitates as a key strategy (9, 20). Moreover, co-culturing enhanced intracellular Cd accumulation (Fig. 2C), potentially by improving cellular membrane fluidity, thus offering a robust barrier against Cd ion penetration (23).

### Cd stress response in bacterial consortia

**Fig 3:**
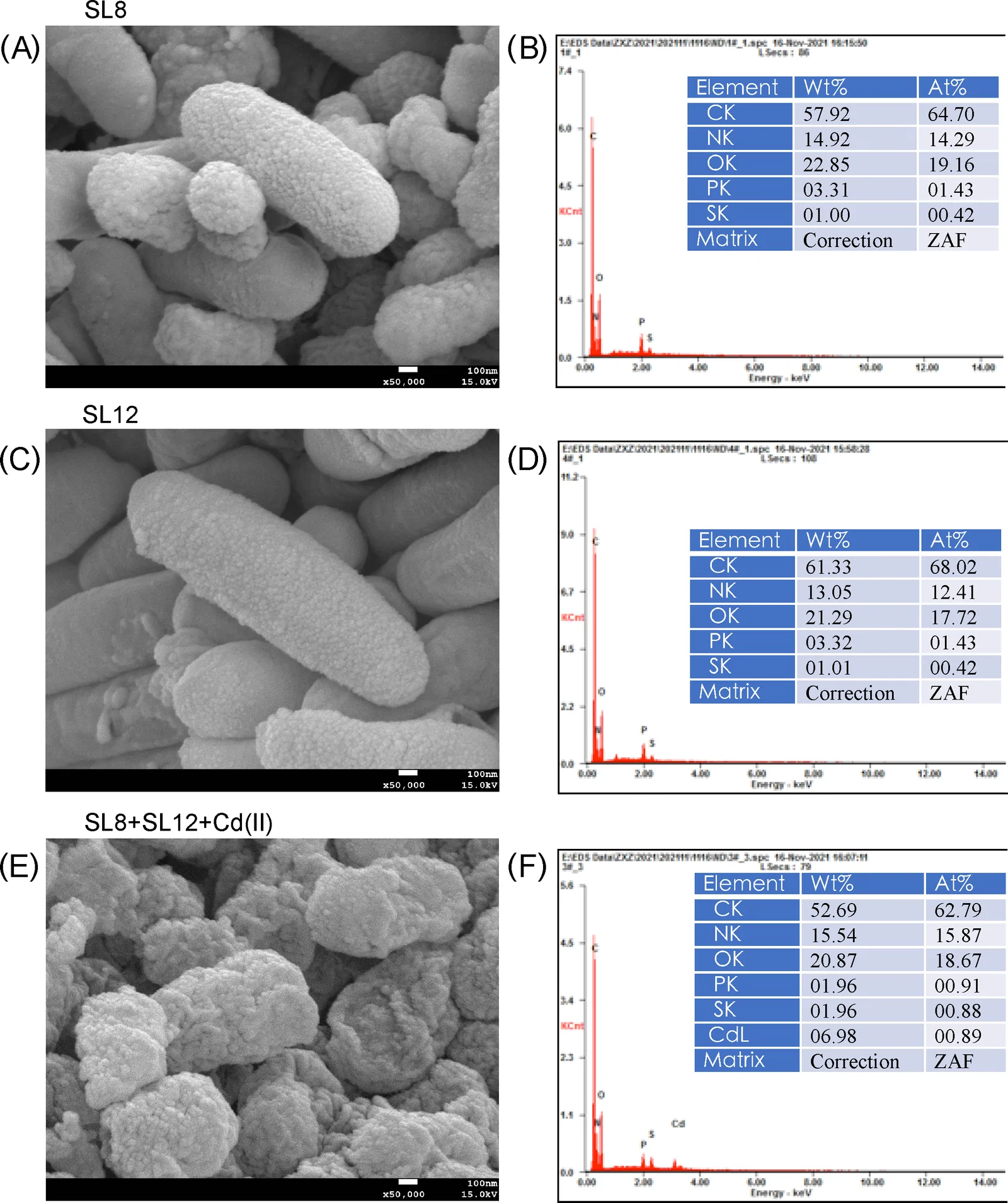
Scanning electron micrographs and energy-dispersive X-ray spectrographs of SL8, SL12, and mixed strains with or without Cd exposure: (**A and B**) SL8 without Cd addition; (**C and D**) SL12 without Cd addition; and (**E and F**) mixed strains of SL8 and SL12 in 100 mg/L Cd^2+^.

**Fig 4:**
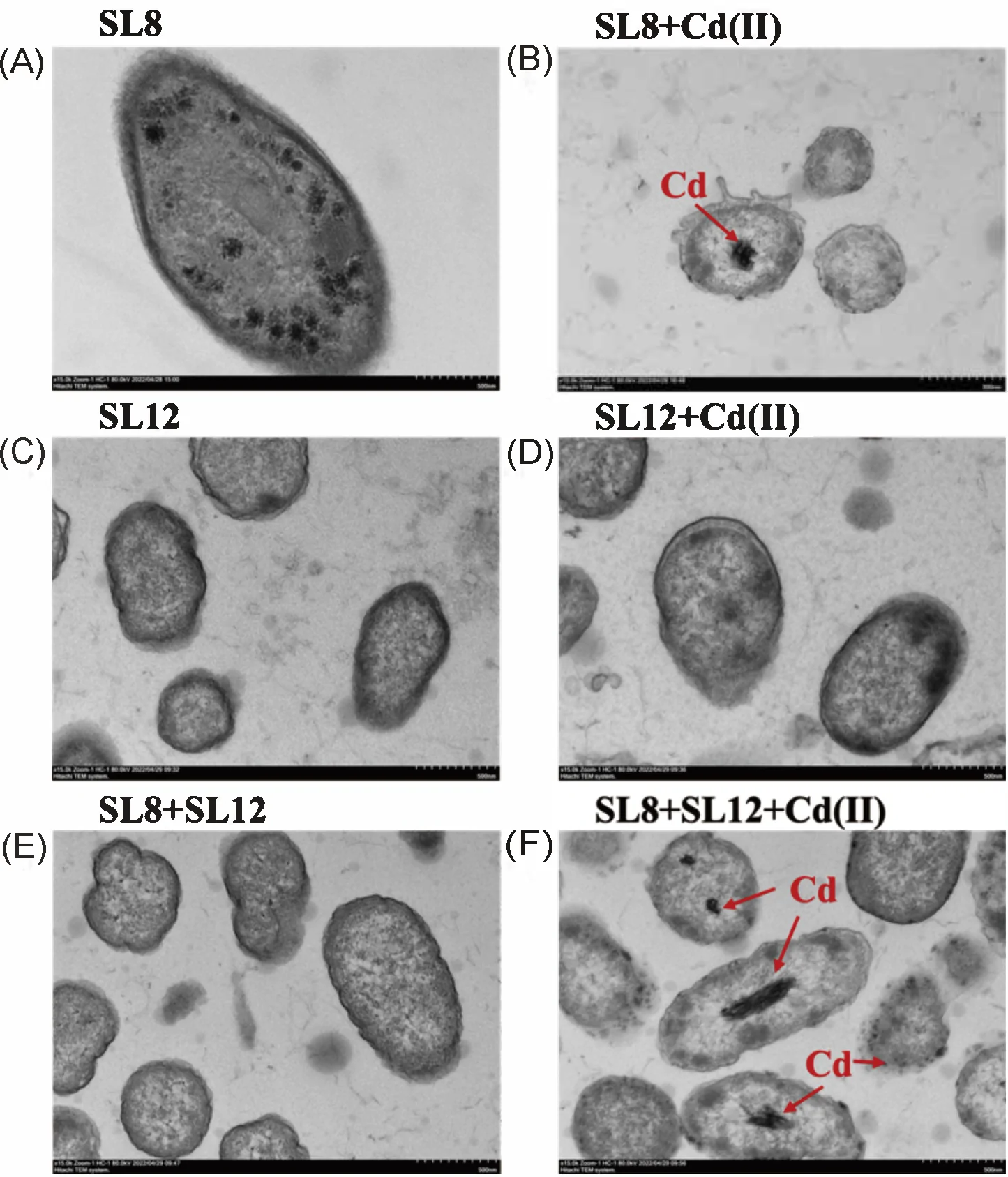
TEM images of strains SL8, SL12, and their cocultures in the absence or presence of 100 mg/L Cd²⁺. (**A**) SL8; (**B**) SL8 with Cd²⁺; (**C**) SL12; (**D**) SL12 with Cd²⁺; (**E**) Coculture of SL8 and SL12; (**F**) Coculture of SL8 and SL12 with Cd²⁺.

The investigation into the characteristics of Cd precipitants formed during incubation of SL8 and SL12 under Cd stress revealed insights into the adaptive mechanisms of these strains. SEM (Fig. 3A, C, and E) and TEM analyses (Fig. 4) showed no significant differences in cell size, cell wall thickness, or cellular outlines between control and Cd-exposed systems for strain SL12. However, SL8 exhibited notable morphological changes. In particular, the thinning and blurring of the cell membrane under Cd stress suggest a stress response that could be pivotal for microorganisms to adapt to toxic environments. This aligns with previous studies (22), which have attributed such morphological changes to a protective strategy that enables bacteria to sustain growth and metabolic activity under metal stress (20).

The response of co-cultured strains, characterized by surface wrinkles, depressions, and deformations without cell membrane rupture, reflects a complex interplay between SL8 and SL12 in adapting to Cd stress. This morphological adaptation, presumably inspired by strain SL8, suggests a communal resilience strategy that could be crucial for survival in heavy metal-contaminated environments. The dense aggregation of cells and abundant crosslinking were observed, possibly resulting from stimulated extracellular polysaccharide (EPS) secretion. This indicates a strategic response to immobilize Cd and prevent Cd migration into the cell bodies. This mechanism, supported by the presence of Cd and S elements on the cell surface as revealed by EDS analysis (Fig. 3B, D, and F), underscores the role of EPS in forming complexes with Cd, consistent with previous research (24).

**Fig 5:**
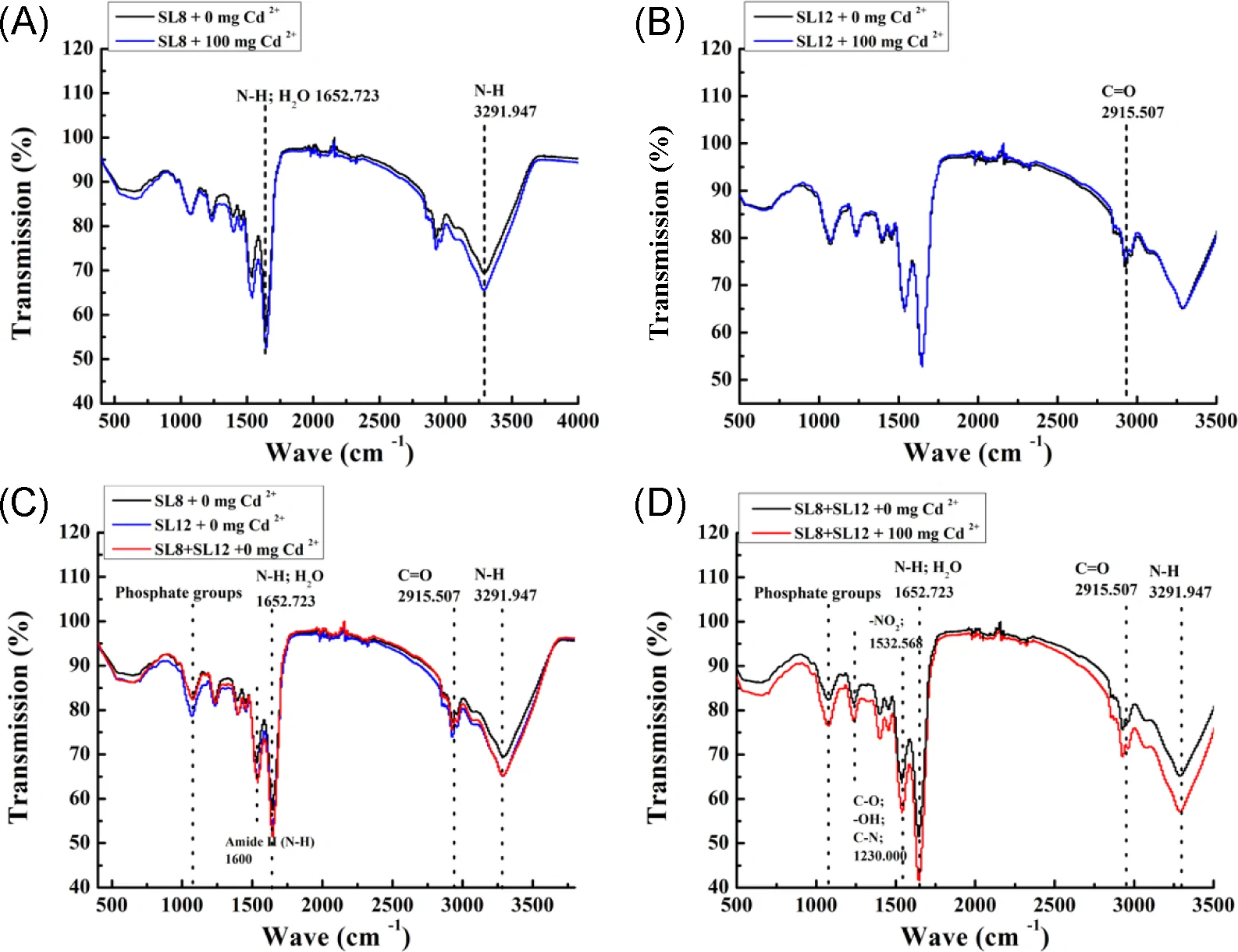
FTIR analysis of precipitants after Cd immobilization by strains SL8, SL12, and their cocultures. (**A–D**) Represent comparisons of different treatments.

**Fig 6:**
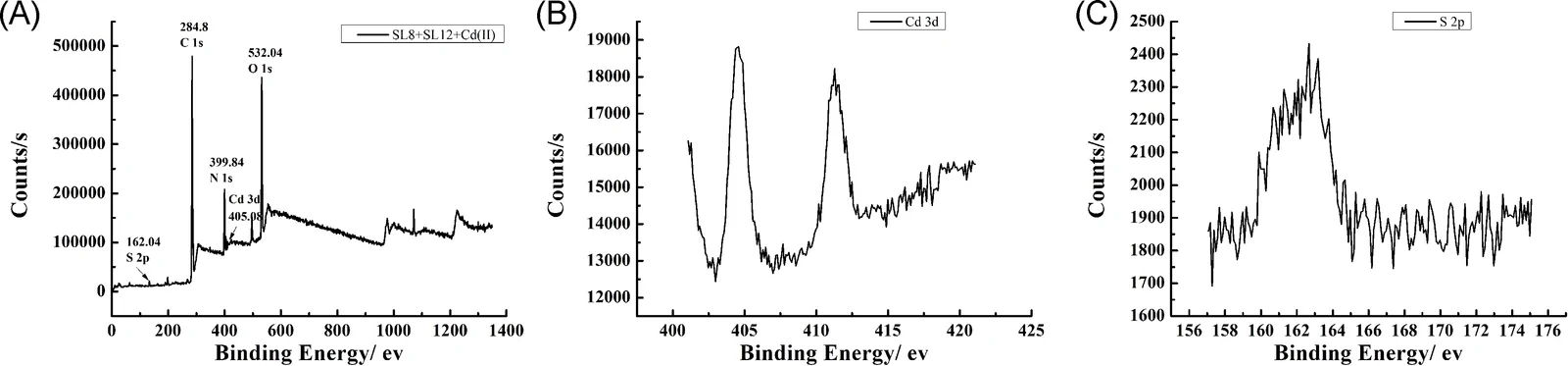
XPS analysis of precipitants after Cd immobilization by cocultured strains of SL8 and SL12. (**A**) Total spectra, (**B**) Cd, and (**C**) S.

The FTIR analysis further elucidated the biochemical mechanisms underlying the enhanced Cd removal performance of the combined strains. The increased abundance of functional groups involved in Cd binding, such as phosphate, amino, and hydroxyl groups, suggested sophisticated biochemical adaptation to Cd stress. Notably, the involvement of N-H bonds in SL8 and amide C=O bonds in SL12 in Cd adsorption highlighted strain-specific responses, while the combined “SL8 + SL12” system exhibited a broadened response spectrum, indicating synergistic effects (Fig. 5). The XPS analysis revealed characteristic peaks of C 1s, N 1s, O 1s, S 2p, and Cd 3d (Fig. 6). The detection of the Cd 3d peak confirmed the presence of CdS and further supported a multi-faceted response to Cd stress. This biochemical strategy, coupled with observed morphological adaptations, highlights the potential of microbial consortia in bioremediation efforts.

This investigation into the stress responses of SL8 and SL12 under Cd exposure illuminated the intricate balance between morphological adaptation and biochemical defense mechanisms. Building on these insights, specific microbial adaptations to cadmium stress were examined, with a focus on metabolic reprogramming and proteomic changes that contribute to the remarkable resilience of these strains. These findings help to bridge molecular response mechanisms with potential applications in bioremediation strategies.

### Microbial adaptation to cadmium stress

**Fig 7:**
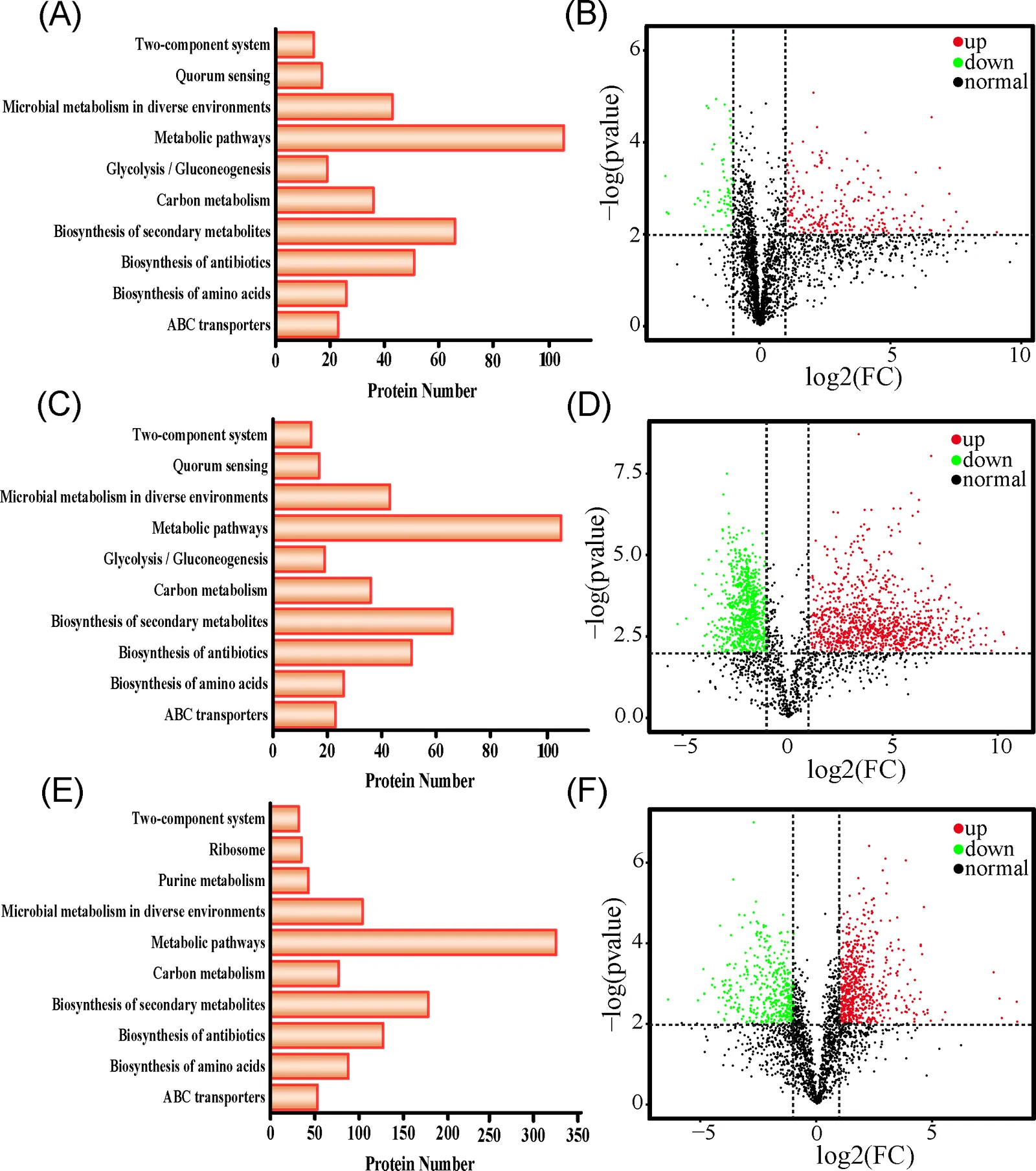
Differentially expressed proteins (DEPs) analysis of group D vs. A, group D vs. B, group D vs. C, respectively (**A**, **C**, and **E**) showed significantly up-regulated DEPs in KEGG enrichment; (**B**, **D**, and **F**) displayed DEPs volcano plots). Proteomic analysis was performed on bacterial samples exposed to high concentrations of Cd^2+^ (100 mg/L): SL8 strains with Cd^2+^ (group A); SL12 strains with Cd^2+^ (group B); SL8 and SL12 cocultured strains without Cd^2+^ (group C); and SL8 and SL12 cocultured strains with Cd^2+^ (group D).

Label-free proteomics analysis revealed significant differential expression of proteins in group D, underscoring a robust response to Cd stress. Specifically, 246, 1,007, and 619 proteins were up-regulated, while 72, 767, and 392 proteins were down-regulated relative to the control groups (Fig. 7B, D, and F). Remarkably, pathways like “Metabolic pathways,” “Biosynthesis of secondary metabolites,” and “Biosynthesis of antibiotics” were predominantly affected (Fig. 7A, C, and E), suggesting that microbes employ enhanced metabolic activity and efflux mechanisms to mitigate Cd toxicity (25).

A deeper comparative analysis of the SL8 and SL12 strains under Cd exposure revealed a sophisticated adaptation at the molecular level. Proteins pivotal to nucleotide transport and metabolism, alongside those involved in defense and inorganic ion transport and metabolism, were notably up-regulated in group D. Key proteins such as exopolyphosphatase, YgiW, Ohr family peroxiredoxin, 3,4-dihydroxy-2-butanone 4-phosphate synthase, ecotin, and hypoxanthine phosphoribosyl transferase (SI, Table S1 available at https://zenodo.org/records/20263325) showed significant alterations, suggesting a refined mechanism for Cd assimilation and sequestration within the cellular matrix, facilitated by specific ion transporters (26). Furthermore, the elevated catalase expression in group D indicated an intrinsic antioxidant response, which likely alleviated the oxidative stress and DNA damage induced by Cd (27). The enhancement of bacterial resistance through the up-regulation of HTH-type transcriptional regulator FarR and chorismate synthase further demonstrated the strategic importance of these proteins in the bacterial efflux system under heavy metal stress (28).

**Fig 8:**
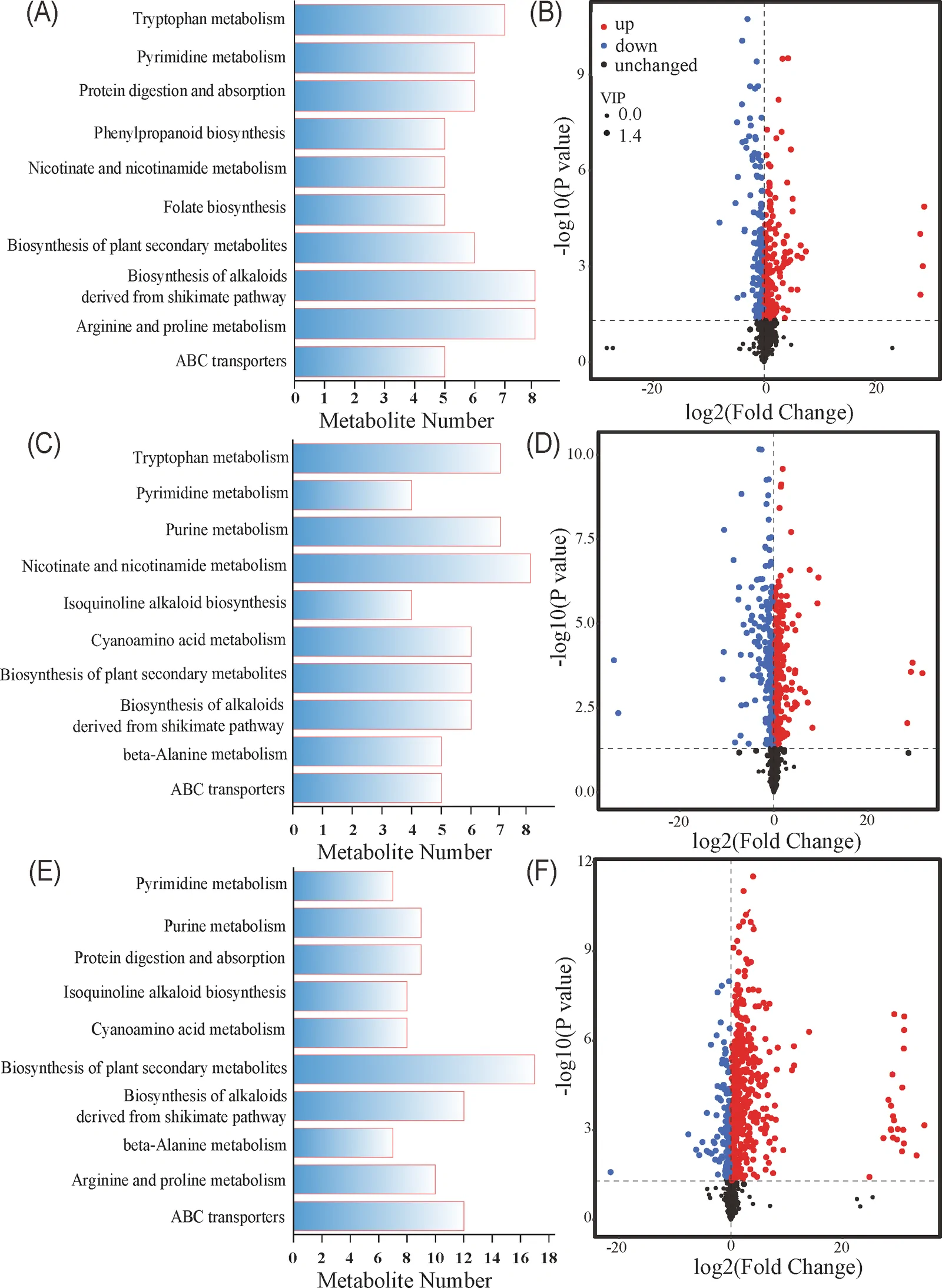
The differential metabolites analysis of groups D vs. A, groups D vs. B and groups D vs. C, respectively. (**A**, **C**, and **E**) show significantly up-regulated differential metabolites in KEGG enrichment; (**B**, **D**, and **F**) display differential metabolite volcano plots. Metabonomic analysis was performed on bacterial samples exposed to high concentrations of Cd^2+^ (100 mg/L): SL8 strains with Cd^2+^ (group A); SL12 strains with Cd^2+^ (group B); SL8 and SL12 cocultured strains without Cd^2+^ (group C); and SL8 and SL12 cocultured strains with Cd^2+^ (group D).

The metabolomic profile (Fig. 8) highlighted significant alterations in the metabolic landscape of the SL8 and SL12 bacterial consortia in response to Cd exposure. This shift was characterized by an increase in 527 metabolites and a decrease in 142 metabolites (Fig. 8F), marking substantial metabolic reprogramming in group D compared to group C. Among these changes, the biosynthesis of plant secondary metabolites, alkaloids derived from the shikimate pathway, and ATP-binding cassette (ABC) transporter activity were most significantly affected (Fig. 8E). The ABC transport system, widely distributed in prokaryotes, plays a crucial role in detoxification by shuttling metal ions and other substrates across cellular membranes, utilizing ATP hydrolysis for energy (29). The up-regulation of this system in group D signified an advanced microbial strategy to reduce intracellular Cd accumulation, enhancing both detoxification and immune protection capabilities.

Further comparison with group A revealed an interesting shift towards the biosynthesis of alkaloids, arginine and proline metabolism, and tryptophan metabolism in group D (Fig. 8A). The involvement of amino acids like proline in combating heavy metal stress underscores their antioxidative role, facilitating chelation of heavy metals into more stable complexes, thereby reducing toxicity (30). Similarly, compared to group B, nicotinate and nicotinamide metabolism exhibited up-regulation in group D, as did tryptophan and purine metabolism (Fig. 8C). The increased tryptophan levels suggested a potential disruption in neurotransmitter synthesis and a redox imbalance under Cd stress (31).

Notably, Cd exposure led to an upsurge in specific dipeptides and signaling molecules implicated in membrane formation within the cocultured system (SI, Table S2 available at https://zenodo.org/records/20263325). These peptides, due to their strong affinity for metal ions, serve as principal Cd-binding agents, facilitating the formation of complexes that enhance the stability and adsorption of Cd ions (32). Additionally, the increase in enzymes related to NADH and NADPH formation indicated augmented, thus presenting a pro metabolic capability geared toward efficient Cd ion transport. The enhancement of NADH/NADPH levels is crucial, as these cofactors play a pivotal role in a myriad of metabolic reactions, including those in the TCA cycle and amino acid oxidation, thereby underscoring the enhanced metabolic adaptability of consortia under Cd stress (33).

Taken together, these results indicate that the SL8 + SL12 consortium enhances Cd immobilization through a multi-faceted molecular synergy. The co-culture achieves more stable alkalinization (Fig. 2G through I), favoring Cd hydroxide precipitation; employs complementary metal-binding groups (N-H from SL8 and amide C=O from SL12; Fig. 5); increases both intracellular Cd accumulation and CdS bioprecipitation (Fig. 2C and 6); and promotes EPS-mediated cell aggregation that traps Cd on cell surfaces (SEM/EDS, Fig. 3 and 4). In parallel, proteomic and metabolomic data reveal unique up-regulation of dipeptides (Cd-chelating agents), NADH/NADPH-related enzymes, and ABC transporters in the consortium (SI, Table S2 available at https://zenodo.org/records/20263325; Fig. 8E). We propose that these synergistic physical, chemical, and metabolic interactions—rather than simple additive effects—underlie the superior Cd removal performance of the SL8 + SL12 consortium.

While our proteomic and metabolomic analyses provide strong correlative evidence for the proposed molecular mechanisms, direct causal validation (e.g., gene knockout) was not performed. Therefore, the up-regulation of specific transporters (e.g., ABC transporters) and antioxidant proteins (e.g., Ohr family peroxiredoxin) should be interpreted as testable hypotheses rather than proven causal factors. Future studies should employ targeted mutagenesis or complementation to confirm the essential role of these candidates in Cd detoxification. Despite these limitations, our integrated multi-omics approach offers a valuable blueprint for understanding and engineering microbial consortia for heavy metal bioremediation.

### Efficacy of co-cultured bacterial strains SL8 and SL12 in cadmium immobilization

Innovative approaches are needed to mitigate the adverse effects of heavy metals, such as Cd, on agricultural productivity and food safety. This study explored the synergistic application of bacterial strains SL8 and SL12 in immobilizing Cd within soil-plant systems, with a particular focus on their impact on cabbage cultivation.

**Fig 9:**
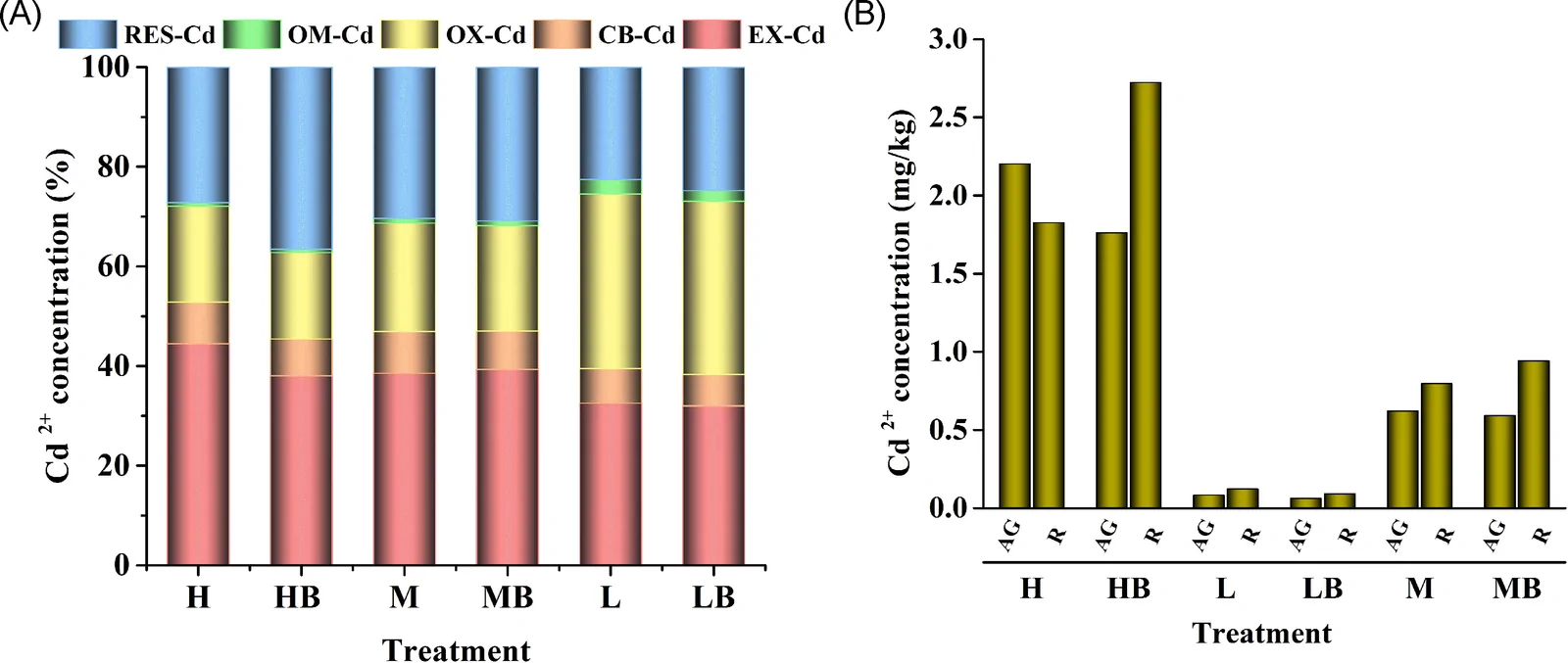
Cadmium fraction distribution in soils (**A**) and total cadmium content in Chinese cabbage roots and aboveground parts (**B**). H; high Cd; HB; high Cd with bacteria; M; medium Cd; MB; medium Cd with bacteria; L; low Cd; LB; low Cd with bacteria; AG; aboveground parts; R; roots.

In this study, soils were artificially spiked with cadmium at concentrations of 0, 5, and 10 mg/kg for pot experiments. The results revealed a discernible decrease of 13% in the bioavailable Cd fractions (exchangeable, carbonate-bound, and Fe-Mn oxidized forms) in the co-cultured strains treatment compared to the control treatment. Concurrently, there was a 33% increase in the non-bioavailable Cd fractions (organic-bound and residual forms), indicating effective immobilization of Cd and reduced phytoavailability (Fig. 9A).

In the context of cabbage cultivation, the application of SL8 and SL12 strains under high Cd stress conditions significantly reduced Cd accumulation in the aboveground parts by 20% (Fig. 9B). This outcome is particularly relevant, given the health risks associated with Cd accumulation in edible plant parts. Interestingly, while the root Cd content increased under high contamination levels, a decrease was observed in low Cd conditions, even with bacterial inoculation. This suggests a complex interaction between bacterial activity, Cd bioavailability, and plant uptake mechanisms, warranting further investigation.

The decrease in Cd uptake in treated cabbage aligned with existing literature, which posited that heavy metal-immobilizing bacteria can effectively reduce metal uptake by vegetables grown in contaminated soils (34, 35). The underlying mechanisms likely involved adsorption and precipitation of Cd by the bacterial strains, which not only restricted Cd availability to plant roots but also impeded its translocation to aerial parts. Furthermore, these strains exerted a beneficial effect on plant growth under Cd stress, as evidenced by increased biomass in treated cabbage. This dual functionality of growth promotion and Cd immobilization highlights the potential of SL8 and SL12 in sustainable agriculture and phytoremediation.

**Fig 10:**
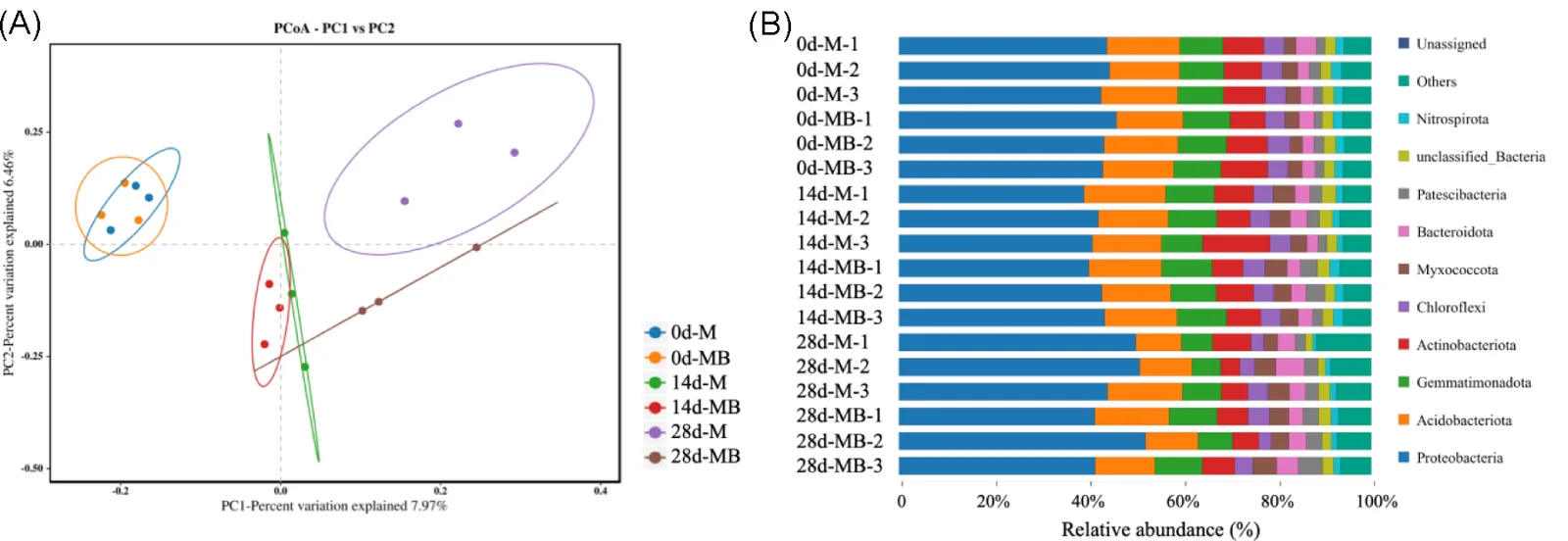
Effect of cocultured strains on rhizosphere soil microbial community structure. (**A**) Principal Coordinate Analysis (PCoA) of bacterial communities at 0-day, 14-day, and 28-day; and (**B**) The cluster diagram based on bacterial community similarity analysis for 0-day, 14-day, and 28-day rhizosphere soils (soil Cd concentration; 5 mg/kg).

Microbial diversity of post-treatment soil revealed no significant alterations (Fig. 10), suggesting that SL8 and SL12 application did not disrupt the soil microbial ecosystem. The stability of dominant microbial groups across treatments indicates that the beneficial effects of these bacterial strains can be achieved without compromising soil microbial health, supporting their ecological compatibility in environmental remediation strategies.

### Conclusion

The synergistic advantage of the co-cultured strains (SL8 and SL12) in Cd immobilization can be attributed to several synergistic mechanisms: (i) enhanced microbial activity: SL8 significantly potentiates the activity of SL12, leading to an increased abundance of functional groups on cell surfaces and the formation of CdS as a key product in the co-culture system; (ii) production of complex metabolites: the co-culture environment facilitates the synthesis of complex compounds, such as dipeptides and signaling molecules, along with direct or indirect metabolites like NADH/NADPH, which interact with mobilized Cd fractions to form more stable complexes; (iii) expression of specific transporters and proteins: specific ion transporters and functional proteins contribute to the adsorption and accumulation of Cd; and (iv) reduced phytoavailability and ecological safety: immobilization of Cd through adsorption and precipitation, lowering its bioavailability and translocation in plants, promoting growth and maintaining soil microbial health. This study expands the repertoire of effective microbial strategies for achieving sustainable agriculture and improved environmental health.

## Data Availability

The 16S rRNA gene sequences of strains SL8 and SL12 have been deposited in the NCBI GenBank database under accession numbers PZ429300 and PZ429301. The proteomic data have been deposited in the iProX partner reposity under accession number IPX0017179000. The metabolomic data have been deposited in the Metabolights database under the accession number MTBLS14477.
